# 

**Published:** 1889-11-09

**Authors:** 


SATURDAY, NOVEMBER 9th, 1889.

**Classification of the Poor in Workhouses** - - 81
**Words of Consolation.**—Paradise - - - - - - 82
**John Chinaman, M.D.** By Dr. J. Bowles Daly - - 83
**Nutrition and Propagation of Flowers and Trees.** By Rev. T. G. Headley - - - - - - - - 84
**The Hospitals Again Vindicated!** - - - - - 84
**The Doctor's Pulpit.**—Life and Living - - - - - 85
**The Home of Rest for Horses** - - - - - 86
**Everybody's Page** - - - - - - - - - - 87
**Notes and News** - - - - - - - - - - 88
**Annotations.**—The Case of Dr. Moon—St. John's Hospital for the Skin—Is Woman the Lesser Man? - - - - 90
**The Practitioner's Outlook and Retrospect:**
Medicine—Antiseptic Drainage of the Lung—Scarlatina Treatment and Results - - - - - - - 91
Surgery—Ununited Fractures - - - - - - - 92
Therapeutics—Quinine—Antipyrin, &c.—Antefebrin - 92
Morphia Injections—Hyoscine as a Sedative - - 93
New Drugs, Appliances, and Things Medical—Temperatures—The Immisch Thermometer—Santha - - 93
**Within the Wards.**—Some Probable Causes of Cancer - 94
**The Hospital Workshop.** XIV.—The Queen's Hospital, Birmingham - - - - - - - - - - 94
**Hypnotism v. Dypsomania—Scraps and Gleanings** 96

EXTRA SUPPLEMENT—THE NURSING MIRROR.

**En Passant.**—A Travelled Story—Short Items—A Lecture to Nurses—Nursing Sisters at Plaistow—An Aberdeen Matron—The Pension Fund—The Asylums Board on Matrons - - - - - - - - - - - xxv
**Three Finches** - - - - - - - - - - xxvi
**The Management of Children.**—I. The Feeding of Infants - - - - - - - - - - - xxvii
**Examination Questions** - - - - - - - - xxvi
**Everybody's Opinion.**—Ladies as District Nurses—A Co-operative Registry Office for Trained Nurses—What Risk We Should Run - - - - - - - - - xxvii
**Notices—Appointments—Notes and Queries** - xxviii
**Presentations—Vacancies** - - - - - - xxviii
**Amusements and Relaxation** - - - - - xxviii

